# A Cross-Sectional Assessment of Quality of Life Among Healthcare Professionals in North-Central Saudi Arabia: Implications for Workforce Well-Being and Policy Development

**DOI:** 10.3390/healthcare14020243

**Published:** 2026-01-19

**Authors:** Ahmad Homoud Al-Hazmi, Fahad Tulayhan M. Alshammari, Ibtisam Qazi, Bashayer Farhan ALruwaili, Doaa Mazen Abdel-Salam, Ashokkumar Thirunavukkarasu

**Affiliations:** 1 Department of Family and Community Medicine, College of Medicine, Jouf University, Sakaka 72388, Saudi Arabia; ftalshammri@moh.gov.sa (F.T.M.A.); immqazi@ju.edu.sa (I.Q.); bfalrwili@ju.edu.sa (B.F.A.); ashokkumar@ju.edu.sa (A.T.); 2 Department of Public Health and Community Medicine, Faculty of Medicine, Assiut University, Assiut 71515, Egypt; doaa.mazen@aun.edu.eg

**Keywords:** quality of life, WHOQOL-BREF, healthcare professionals, physical, environmental, social

## Abstract

**Background and Objectives:** Quality of life (QoL) among healthcare professionals (HCPs) is a critical determinant of workforce performance and patient care. Therefore, the present study aimed to assess QoL and its determinants among HCPs in the Hail region, Saudi Arabia. **Methods:** In this cross-sectional study, data were collected from 388 HCPs from multiple healthcare facilities using the WHOQOL-BREF questionnaire. The survey was conducted from August 2025 to October 2025. Convenience sampling was used, and QoL domain scores were calculated according to WHO guidelines. We applied Spearman’s correlation test to assess correlations across domains and logistic regression to identify factors associated with individual and overall QoL. **Results:** Among the HCPs studied, overall QoL had a median score of 80, while the physical, psychological, social, and environmental domains showed moderate scores with considerable variability. We found a significant positive correlation between the various QoL domains (*p* = 0.001). Non-Saudi nationals (*p* = 0.010) and participants with chronic diseases (*p* = 0.032) reported significantly lower overall QoL. Furthermore, age group, work experience, HCPs category, work setting, nationality, and the presence of chronic disease were significant predictors across multiple QoL domains. **Conclusions:** The findings highlight the need for targeted workplace and health support interventions to manage the mental and physical health of HCPs, particularly for non-Saudi HCPs and those with chronic conditions, through tailored training, education, and lifestyle-based support programs.

## 1. Introduction

Quality of life (QoL) has gained increasing recognition as a fundamental concept in public health, clinical practice, and healthcare management [1,2]. According to the World Health Organisation (WHO), QoL is a person’s assessment of their place in life in relation to their objectives, standards, expectations, and worries, as well as the culture and value systems in which they live [3]. In the context of healthcare, QoL is not just a measure of personal satisfaction; it is closely linked to healthcare performance and the provision of safe, effective, and compassionate care. With the growing number of patients, the increasing burden of chronic illnesses, and the changing expectations of patients as pressure mounts on the healthcare system worldwide, the concept of QoL among healthcare professionals (HCPs) has become more significant [2,4,5]. HCPs are exposed to specific work-related stressors that can disrupt their QoL. Their duties frequently include long and irregular working hours, shift work, a large number of patients, and constant emotional distress, suffering, and life-threatening experiences. These challenges have a cumulative effect, which may cause burnout, emotional exhaustion, loss of empathy, and poor job performance, increasing the risk of medical errors, reducing productivity, diminishing patient satisfaction, and increasing turnover intentions. The promotion of HCPs’ well-being is thus necessary not only for their well-being but also for the high standards of patient care and the performance of the healthcare system [4,6,7].

The healthcare industry in Saudi Arabia has experienced substantial growth and change following the national Vision 2030 agenda. Key reforms include health service modernization, growth in digital health, redesign of service provision, and strategic investments in human capital. These efforts are expected to enhance access to quality care and enhance the overall effectiveness of the healthcare system [8,9,10]. Nevertheless, these fast changes have imposed new burdens on HCPs, who are now to adjust to technological advances, changing clinical practice, and rising service expectations. Previous studies have documented differences in levels of burnout, occupational stress, and work-related strain among HCPs [11,12]. Education levels, residing areas, heavy workload, and inadequate time for professional development have been reported as factors contributing to lower QoL across various Saudi settings [11,13,14].

Potential problems for HCPs in other regions, such as Hail, include variability in patient numbers and resources, and a reduced number of specialists or a lower level of clinical training compared to large cities such as Riyadh, Jeddah, and Dammam [15,16,17]. In addition, the Hail region is characterized by wide geographic dispersion, a relatively smaller healthcare workforce, and limited access to advanced professional training opportunities, all of which may intensify work-related pressures and influence QoL among HCPs [16,17,18]. Despite growing evidence on QoL and workforce well-being in major urban centers of Saudi Arabia, empirical data focusing on HCPs in peripheral and less urbanized regions such as Hail remain limited, resulting in an underrepresentation of regional workforce challenges in the existing literature [13,15,18]. Moreover, employee hiring and retention remain a burning issue in most healthcare facilities in the region, affecting job satisfaction and overall QoL. Although these facts are present, there is a significant gap in empirical data on QoL among HCPs in Hail, leading to a lack of evidence-based planning and workforce policymaking. However, as the national focus on workforce welfare increases, the available evidence is unevenly distributed. Addressing this gap is essential for supporting the goals of the Health Sector Transformation Program under Vision 2030, which emphasizes building a resilient, motivated, and highly skilled healthcare workforce [10].

To develop regional strategies to improve the workplace situation, reliable, context-related QoL data are required [18,19]. Given the constant changes in healthcare delivery in Saudi Arabia, it is necessary to continuously monitor epidemiological data on workforce well-being to inform evidence-based planning. Therefore, the present study aimed to assess QoL and its determinants among HCPs in the Hail region, Saudi Arabia.

## 2. Materials and Methods

### 2.1. Study Description

The present study employed a cross-sectional design and was conducted from August 2025 to October 2025 in the Hail region of Saudi Arabia. The Hail region, located in north-central Saudi Arabia, presents an important yet understudied context for examining QoL among HCPs. The region includes a mix of tertiary hospitals, general hospitals, and primary healthcare centers serving both urban residents and widely dispersed rural communities. The present study included all HCPs from all types of healthcare facilities, both Saudi and non-Saudi nationalities, who were directly involved in patient care and had been working in their respective healthcare facilities for at least 6 months. We excluded those on long leave, on vacation, interns, trainees, and students, as well as HCPs who were only involved in administrative work.

### 2.2. Sampling Description

We used an online sample size calculator to estimate the required sample size [20]. With a 50% expected proportion (*p* = 0.5, q = 1 − *p*), a 95% confidence interval, and a 5% margin of error, the minimum required HCPs to obtain a valid conclusion was 384. The research team used convenience sampling to recruit participants. HCPs who were available during the data collection period and met the eligibility criteria were invited to participate. This approach was selected because of practical limitations in reaching various healthcare institutions in the Hail region and the need to take appropriate measures to involve representatives of different professional groups. Although convenience sampling may not provide a complete representation of the target population, it is widely used in cross-sectional surveys of the health workforce and is suitable for exploratory evaluations of QoL within operational healthcare settings. Furthermore, this sampling method was used due to the difficulty in obtaining a comprehensive sampling frame and operational constraints related to participant availability within routine. To reduce selection bias, participants were recruited from diverse professional categories and multiple healthcare settings within the region.

### 2.3. Ethical Considerations

We conducted this study in accordance with the Declaration of Helsinki. For this study, ethical clearance was obtained from the IRB, Hail Health Cluster (Log no: 2025-76, dated 23 July 2025). Participation was voluntary, and informed consent was obtained from all participants prior to data collection.

### 2.4. Data Collection Steps

After obtaining the required administrative approvals, the data collectors approached the eligible HCPs as mentioned earlier. The data collectors briefed the study participants and obtained informed consent for voluntary participation. Those who agreed to participate in the study were asked to complete a Google form based on the current research’s data collection form, which consisted of two sections. Eligible participants were approached personally at their workplace, and responses were recorded on-site using the data collectors’ personal electronic devices. The first section inquired about the background characteristics of HCPs, including gender, age group, marital status, nationality, educational qualification, professional category (e.g., physicians, nurses, pharmacists, laboratory technicians, and others), years of work experience, work setting (primary care, general hospital, or specialty hospital), and presence of chronic diseases. The second section utilized the WHOQOL-BREF, which is widely used internationally and is suitable for assessing multidimensional QoL among HCPs [3,11,12]. Nonetheless, we used the same data collection tool with 33 HCPs to assess clarity, cultural adaptability and acceptability. All participants agreed that the tool is socially acceptable and easy to understand, and no modifications were made to the original tool based on the pilot study participants’ feedback. The pilot study participants were excluded from the final study sample. This instrument includes 26 items that assess four major domains of QoL: physical health (7 items), psychological health (6 items), social relationships (3 items), and environmental health (8 items). Specifically, the physical health domain assesses energy, mobility, pain, sleep, and daily activities; the psychological domain evaluates positive and negative feelings, self-esteem, and cognitive functions. The social relationships domain examines personal relationships and social support, while the environmental domain covers financial resources, safety, healthcare access, and physical living conditions. Two additional items assess overall QoL and general health perception.

The study’s scoring was performed according to the official WHO guidelines, such as reverse scoring for negatively framed questions. Raw scores for each domain were calculated by summing item scores belonging to that domain. Furthermore, we converted raw domain scores from 0–100 using the following transformation formula: Transformed Domain Score = ((Actual Raw Score − Lowest Possible Raw Score)/(Possible Raw Score Range)) × 100. This was performed for interpretation and comparison. Therefore, higher scores indicate better QoL across physical, psychological, social, and environmental domains.

### 2.5. Data Analysis

The research team used the Statistical Package for the Social Sciences (SPSS) version 21.0 (IBM Corp., Armonk, NY, USA) for data export, coding, and further analysis (Please find the Supplementary File S1—WHO QoL Final for more details). The continuous data did not meet the normality assumption, as assessed by the Shapiro–Wilk test. We presented the categorical data as frequencies and proportions, and the continuous data as medians and interquartile ranges (IQRs). Furthermore, nonparametric tests (Spearman’s rank correlation) were used to find correlation across the domains. For regression analyses, each QoL domain was dichotomized into “low” and “high” categories based on median splits. QoL domain scores were dichotomized using median values to facilitate comparative analysis, given the ordinal nature of the WHOQOL-BREF and the non-normal distribution of domain scores. Categorization of QoL scores based on measures of central tendency, including mean or median cut-offs, has been widely applied in QoL and public health research when distributional assumptions required for parametric analyses are not met [21,22,23,24]. In the binary logistic regression analysis (enter method), gender, age group, marital status, nationality, educational level, professional category, years of work experience, work setting, and presence of chronic disease were used as independent variables. Adjusted odds ratios (AORs) with 95% confidence intervals (CI) were reported. A *p*-value < 0.05 was considered statistically significant.

## 3. Results

Table 1 revealed the sociodemographic characteristics of the participants. Most HCPs were females (50.5%), single (60.3%), Saudi nationals (77.3%), and working in general hospitals (46.1%). Regarding educational level, 48.2% of participants held a bachelor’s degree. Forty-four percent of HCPs had less than five years of work experience, while 32.2% had between five and ten years of experience.

Table 2 shows overall QoL and domain-specific QoL scores. The overall QoL domain shows the highest median score (80). Among the specific domains, the environment domain had a slightly higher median (68) compared to the physical health (66), psychological (67), and social relationships (67) domains. The wide range across all domains (20–100) indicates substantial variability in participants’ responses, reflecting diverse experiences and perceptions of QoL within the study population.

The data in the present study did not satisfy the assumption of normality; therefore, Spearman’s rank correlation test was applied, revealing that all correlation coefficients were statistically significant at *p* < 0.001 and showed a positive correlation between the various QoL domains (Table 3).

Table 4 presents a binomial logistic regression analysis of predictors of the physical and psychological health domains. It revealed that age and work experience were significantly associated with the physical health domain. Participants aged 44 or older had higher odds of having low physical health (OR = 2.73, CI: 1.54–4.83, *p* = 0.001). However, participants with 11 to 15 years of work experience were more likely to have good physical health (OR = 0.48, CI: 0.24–0.98, *p* = 0.046). Table 4 also showed that age, healthcare profession, work experience, and work setting were significantly associated with the psychological health domain. HCPs older than 44 years (OR = 1.83, CI: 1.03–3.28, *p* = 0.041), nurses (OR = 1.70, CI: 1.14–2.55, *p* = 0.029), and those with work experience of 5 to 10 years (OR = 3.80, CI: 1.47–9.87, *p* = 0.006) had higher odds of having low psychological health. However, those working in specialty hospitals reported better psychological health (OR = 0.44, CI: 0.25–0.79, *p* = 0.006).

Table 5 revealed logistic regression analysis of the predictors of social relationships and environmental domains. Age and education were significantly associated with the social relationship domain. Participants aged 44 or older had higher odds of having low social relationships (OR = 1.94, CI: 1.10–3.46, *p* = 0.023). HCPs holding an MD, PhD, or Saudi Board certification were significantly more likely to report social relationships (OR = 0.29, 95% CI: 0.12–0.72, *p* = 0.007). In addition, work experience, work setting, and the presence of chronic diseases were significantly associated with the environmental domain. Participants with 5 to 10 years of work experience were less likely to be satisfied with environmental conditions (OR = 3.36, CI: 1.35–5.34, *p* = 0.009). In contrast, working in specialty hospitals (OR = 0.43, 95% CI: 0.25–0.77, *p* = 0.004) and having chronic diseases (OR = 0.47, 95% CI: 0.28–0.80, *p* = 0.005) were significantly associated with higher environmental QoL.

Table 6 presents the predictors associated with overall QoL. Non-Saudi personnel (OR = 2.43, CI: 1.23–3.82, *p* = 0.010) and participants with chronic diseases (OR = 1.75, CI: 1.15–2.63, *p* = 0.032) reported significantly lower overall QoL.

## 4. Discussion

It is essential to ensure that HCPs maintain a satisfactory level of QoL, as they represent a vital element of the healthcare system. Their well-being is essential for providing high-quality patient care and ensuring safer health outcomes. Policymakers and stakeholders should strive to enhance the QoL of HCPs to boost their performance and increase their job satisfaction. Organizational-level interventions, workforce wellbeing, and policy development should form an integrated continuum, where effective implementation at the organizational level informs policy, and policy, in turn, reinforces systemic improvements in workforce wellbeing. The present study is aimed at assessing QoL among HCPs in North Central Saudi Arabia and identifying its associated factors. In the present study, the overall QoL domain shows the highest median score, indicating that participants generally reported a moderate overall perception of their QoL. Among the specific domains, the environmental domain had a slightly higher median compared to the physical health, psychological, and social relationships domains, suggesting relatively better satisfaction with environmental aspects in this study, in agreement with a study conducted in Romania, which showed that HCPs reported high QoL scores across all four dimensions, particularly in environmental health during the COVID-19 pandemic [25].

Conversely, a recent study revealed that Pakistani HCPs had higher mean scores for the psychological and social domains compared to the physical and environmental domains. This reveals good individual relationships and great social support. This difference may be explained by the timing of the surveys, as the Pakistani study was conducted in December 2020, less than a year after COVID-19 was declared a pandemic [26]. A comparison between the current study’s findings and those reported in earlier studies from low- and middle-income countries reveals the following: a study conducted in the Gaza Strip revealed a moderate overall QoL level among HCPs [23]. A population-based survey in Syria conducted by Al Houri et al. among 700 HCPs showed that half of the respondents had a good QoL [27]. Another study in Saudi Arabia aimed to assess HCPs’ QoL and associated factors and reported that most participants were satisfied with the quality of their living conditions, including their workplaces and environmental health [13]. A similar population-based study among nurses working at King Abdulaziz University Hospital, Jeddah, Saudi Arabia, found that they had higher QoL, with increasing salaries and shorter shift times being the most important determinants [28]. However, a cross-sectional study of HCPs in Uganda reported that participants had low overall work-related QoL [29].

The present study showed that HCPs with 5 to 10 years of work experience had lower QoL in the psychological and environmental domains, in agreement with a study conducted in the Gaza Strip [23]. Italia et al. showed that greater work experience was negatively associated with good QoL [30], whereas Anshasi et al. demonstrated that longer work experience adversely affected only the physical domain of QoL [31]. This can be explained by the fact that less senior staff are less vulnerable to work-related overload and stress.

Although the nursing workforce has expanded steadily over recent decades, nurses have faced increasing workloads in recent years, largely driven by rising service demand and inadequate staffing ratios [32], which has negatively affected their QoL [33]. In addition, nurses are exposed to numerous stressors, including unfavorable work environments, fatigue, inadequate resources, heightened infection risk, and low wages [34]. The present study showed that HCPs who were nurses had lower psychological health than those in other professions. This agrees with available data reports, which showed a moderate-to-low QoL among nursing staff in Saudi hospitals [35,36,37].

A previous study indicated that HCPs over the age of 40, having more work experience and a deeper understanding of their job and working conditions, tended to report a better QoL [38]. This contradicts the results of the present study, which showed that HCPs aged 44 or older reported poorer physical and psychological health, as well as diminished social relationships. This unexpected association highlights the importance of considering cumulative occupational and age-related factors when interpreting QoL outcomes among HCPs [39,40]. The contradiction in the present may be explained by several factors. As HCPs increase in their age, they often face greater physical strain, chronic health issues, and fatigue from years of demanding work, which can negatively influence their physical and psychological well-being. In addition, increased job responsibilities, stress, and potential burnout may contribute to reduced mental health. Social relationships may decline due to work–life imbalance, limited time for social interactions, or emotional exhaustion accumulated over the years.

The absence of significant gender differences found in the present study across several QoL domains may reflect relatively similar job roles, workload distribution, and institutional policies for male and female HCPs within the study setting, which may attenuate gender-based disparities reported in other contexts. However, our study findings are in align with some other studies [23,41]. Populations with higher education levels often prioritize their well-being and actively engage in activities that enhance their overall health. Furthermore, Individuals with higher education are more inclined to adopt healthier lifestyles and practice preventive measures, leading to an overall improvement in QoL [42]. In the present study, highly educated HCPs had better social relationships compared to others.

The presence of chronic diseases not only leads to treatment challenges, increased usage of medicine, and a heavier burden of disease, but also significantly influences the QoL of the patients [43]. Studies have shown that people with chronic diseases reported significantly lower perceived QoL [44,45], which is consistent with the results of the present study. In the present study, we found that overall QoL and environmental domain is significantly associated with the patients with chronic diseases. Among HCPs, this association may reflect the combined impact of ongoing disease management, work-related demands, and concerns about long-term health, which together can adversely affect perceived well-being even among HCPs [46,47].

This study highlighted that the workplace was an important factor associated with various domains of QoL. Working in specialty hospitals had a significant positive effect on the psychological and environmental domains of the QoL, consistent with a study conducted in the Gaza Strip [45]. In contrast, a systematic review of ten studies reported that primary healthcare nurses generally demonstrated a high level of professional QoL [48]. The present study revealed that non-Saudi HCPs had lower Overall QoL compared to Saudis. This disparity may be attributed to several factors: many non-Saudi HCPs are employed on limited-term contracts, which can lead to instability and stress; they often receive lower salaries or fewer benefits; and many live away from their families, resulting in reduced social support. In addition, they may face limited opportunities for career advancement and frequently experience higher workloads and job strain.

The current study offers a comprehensive examination of QoL and its associated factors among HCPs in North Central Saudi Arabia, with significant public health implications for policies that help HCPs navigate systemic challenges and build sustainable healthcare systems. However, the present study has several limitations. First, because the study is observational and uses a cross-sectional design, it cannot establish causal relationships. Second, recall bias can occur because the data are self-reported. Third, although respondents were assured anonymity and confidentiality, and no identifying information was collected, social desirability and other response biases cannot be completely ruled out. Fourth, QoL among HCPs from refugee families may be lower than that of those who were not, and this should be taken into consideration in further research. Next, the use of convenience sampling may have introduced selection bias, which could have influenced the observed associations between participant characteristics and QoL domains and it may limit the generalizability of the findings beyond the study population. Moreover, the absence of organizational and workplace-related variables, such as salary, workload, shift patterns, and institutional support, limits a more comprehensive understanding of factors influencing QoL and may partially explain the observed differences across subgroups.

## 5. Conclusions

The present study showed that HCPs generally reported a moderate overall QoL in North Central Saudi Arabia. Age, work experience, workplace, and education were associated with different domains of QoL. Furthermore, non-Saudi and HCPs with chronic diseases experienced lower overall QoL. From a policy perspective, targeted occupational health programs and institution-based mental health support services should be prioritized to promote both physical and psychological well-being among HCPs. Enhanced support should be given priority through special training and education interventions, especially to non-Saudi HCPs, as well as those with chronic diseases, along with tailored workforce policies addressing occupational stress, job security, and access to support services for expatriate healthcare workers. Finally, future research should consider qualitative studies to explore the contextual and experiential aspects of each QoL domain in greater depth.

## Figures and Tables

**Table 1:** Socio-demographic characteristics of HCPs (*n* = 388).

Variable	Frequency	Proportion
Gender		
Male	192	49.5
Female	196	50.5
Age groups, year		
Less than 34	101	26.0
35 to 43	142	36.6
More than 44	145	37.4
Marital status		
Single	234	60.3
Married	84	21.6
Divorce/widowed	70	18.1
Nationality		
Saudi	300	77.3
Non-Saudi	88	22.7
Education		
Diploma	59	15.2
Bachelor	187	48.2
Master	79	20.4
MD/PhD/Saudi Board	63	16.2
HCPs’ category		
Physicians and dentists	71	18.3
Nurse	143	36.9
Pharmacists	82	21.1
Lab technicians	53	13.6
Other	39	10.1
Work experience		
Less than 5 years	93	44.0
5 to 10 years	125	32.2
11 to 15 Years	97	25.0
More than 15 years	73	18.8
Work Setting		
PHC	94	24.2
General hospital	179	46.1
Specialty	115	29.6
Presence of chronic disease		
No	276	71.1
Yes	112	28.9

**Table 2:** Overall QoL and QoL scores of HCPs across various domains.

Domain	Median (IQR)	Min–Max
Physical Health	66 (54–77)	23–100
Psychological	67 (53–80)	20–100
Social Relationships	67 (40–80)	20–100
Environment	68 (50–78)	20–100
Overall QoL	80 (70–90)	20–100

**Table 3:** Spearman correlations between various domains of QoL.

	Physical Health	Psychological	Social Relationships	Environment
Physical Health	-	0.650 (0.001)	0.683 (0.001)	0.666 (0.001)
Psychological	0.650 (0.001)	-	0.747 (0.001)	0.839 (0.001)
Social Relationships	0.683 (0.001)	0.747 (0.001)	-	0.852 (0.001)
Environment	0.666 (0.001)	0.840 (0.001)	0.839 (0.001)	-

**Table 4:** Predictors associated with the physical and psychological health of HCPs.

Variables	Total *n* = 388	Physical Health				Psychological			
		Low *n* = 182	High *n* = 206	Adjusted Odds Ratio AOR (95% CI)	*p*-Value	Low *n* = 175	High *n* = 213	AOR (95% CI)	*p*-Value
Gender									
Male	192	93	99	Ref		83	109	Ref	
Female	196	89	107	0.849 (0.53–1.37)	0.501	92	104	1.078 (0.66–1.76)	0.763
Age groups, year									
Less than 34	101	45	56	Ref		34	67	Ref	
35 to 43	142	53	89	0.983 (0.43–2.27)	0.968	65	77	1.815 (0.79–4.17)	0.160
More than 44	145	84	61	2.730 (1.54–4.83)	0.001	76	69	1.833 (1.03–3.28)	0.041
Marital status									
Single	234	111	123	Ref		111	123	Ref	
Married	84	29	55	1.442 (0.78–2.67)	0.245	28	84	0.876 (0.47–1.64)	0.678
Divorce/widowed	70	42	28	2.344 (0.96–3.76)	0.053	36	70	0.702 (0.28–1.76)	0.449
Nationality									
Saudi	300	143	157	Ref		137	163	Ref	
Non–Saudi	88	39	49	0.748 (0.40–1.39)	0.357	38	50	0.936 (0.50–1.76)	0.838
Education									
Diploma	59	30	29	Ref		23	36	Ref	
Bachelor	187	82	105	0.635 (0.23–1.75)	0.380	86	101	1.688 (0.61–4.71)	0.316
Master	79	41	38	1.011 (0.42–2.41)	0.981	42	37	0.999 (0.41–2.41)	0.999
MD/PhD/Saudi Board	63	29	34	0.650 (0.27–1.57)	0.337	24	39	0.730 (0.30–1.76)	0.483
HCPs’ category									
Physicians and dentists	71	32	39	Ref		25	46	Ref	
Nurse	143	74	69	1.219 (0.47–3.19)	0.687	77	66	1.701 (1.14–2.55)	0.029
Pharmacists	82	33	49	0.809 (0.37–1.79)	0.602	31	51	0.689 (0.31–1.55)	0.369
Lab technicians	53	26	27	1.462 (0.63–3.37)	0.372	27	26	1.464 (0.62–3.43)	0.381
Other	39	17	22	0.943 (0.38–2.35)	0.901	15	24	0.658 (0.26–1.68)	0.383
Work experience									
Less than 5 years	93	30	63	Ref		21	72	Ref	
5 to 10 years	125	66	59	1.159 (0.47–2.86)	0.749	67	58	3.807 (1.47–9.87)	0.006
11 to 15	97	55	42	0.486 (0.24–0.98)	0.046	57	40	0.742 (0.36–1.52)	0.413
More than 15 years	73	31	42	0.529 (0.26–1.06)	0.072	30	43	0.640 (0.32–1.29)	0.210
Work Setting									
PHC	94	34	60	Ref		40	54	Ref	
General hospital	179	97	82	1.343 (0.68–2.86)	0.390	95	84	0.510 (0.26–1.01)	0.054
Specialty	115	51	64	0.693 (0.40–1.21)	0.194	40	75	0.446 (0.25–0.79)	0.006
Presence of chronic disease-DM									
No	276	122	154	Ref		129	147	Ref	
Yes	112	60	52	1.255 (0.75–2.11)	0.391	46	66	0.752 (0.44–1.27)	0.290

**Table 5:** Predictors associated with the social relationships and environment domains of HCPs.

Variables	Total *n* = 388	Social Relationships				Environmental Domain			
		Low *n* = 181	High *n* = 207	AOR (95% CI)	*p*-Value	Low *n* = 192	High *n* = 196	AOR (95% CI)	*p*-Value
Gender									
Male	192	87	105	Ref		95	97	Ref	
Female	196	94	102	1.083 (0.67–1.76)	0.745	97	99	1.003 (0.61–1.64)	0.991
Age groups, year									
Less than 34	101	37	64	Ref		37	64	Ref	
35 to 43	142	68	74	1.917 (0.85–4.32)	0.117	73	69	1.651 (0.73–3.76)	0.233
More than 44	145	76	69	1.947 (1.10–3.46)	0.023	82	63	1.712 (0.95–3.07)	0.072
Marital status									
Single	234	117	117	Ref		132	102	Ref	
Married	84	27	57	1.005 (0.54–1.86)	0.987	25	59	0.777 (0.42–1.44)	0.424
Divorce/widowed	70	37	33	1.469 (0.61–3.55)	0.392	35	35	1.485 (0.61–3.60)	0.381
Nationality									
Saudi	300	148	152	Ref		156	144	Ref	
Non–Saudi	88	33	55	0.596 (0.32–1.11)	0.104	36	52	0.776 (0.42–1.45)	0.427
Education									
Diploma	59	27	32	Ref		33	26	Ref	
Bachelor	187	89	98	0.540 (0.19–1.48)	0.231	96	91	0.521 (0.19–1.44)	0.209
Master	79	45	34	0.419 (0.61–3.55)	0.052	40	39	0.453 (0.18–1.09)	0.077
MD/PhD/Saudi Board	63	20	43	0.295 (0.12–0.72)	0.007	23	40	0.494 (0.20–1.20)	0.121
HCPs category									
Physicians and dentists	71	28	43	Ref		31	40	Ref	
Nurse	143	74	69	0.896 (0.35–2.33)	0.822	80	63	0.652 (0.24–1.74)	0.393
Pharmacists	82	34	48	1.111 (0.52–2.46)	0.795	39	43	0.782 (0.35–1.77)	0.557
Lab technicians	53	28	25	1.665 (0.73–3.82)	0.228	26	27	1.101 (0.47–2.58)	0.825
Other	39	17	22	0.758 (0.31–1.88)	0.550	16	23	0.699 (0.27–1.79)	0.456
Work experience									
Less than 5 years	93	27	66	Ref		27	66	Ref	
5 to 10 years	125	67	58	1.792 (0.74–4.36)	0.197	69	56	3.362 (1.35–5.34)	0.009
11 to 15	97	55	42	0.649 (0.32–1.32)	0.233	54	43	1.113 (0.54–2.30)	0.773
More than 15 years	73	32	41	0.625 (0.31–1.26)	0.186	42	31	1.202 (0.59–2.44)	0.610
Work Setting									
PHC	94	40	54	Ref		46	48	Ref	
General hospital	179	97	82	0.802 (0.41–1.56)	0.517	103	76	0.530 (0.27–1.04)	0.065
Specialty	115	44	71	0.634 (0.37–1.10)	0.106	43	72	0.437 (0.25–0.77)	0.004
Presence of chronic disease									
No	276	137	139	Ref		146	127	Ref	
Yes	112	44	68	0.599 (0.36–1.01)	0.054	43	69	0.472 (0.28–0.80)	0.005

**Table 6:** Predictors associated with the overall QoL of HCPs.

Variables	Total *n* = 388	Overall QoL			
		Low *n* = 154	High *n* = 234	AOR (95% CI)	*p*-Value
Gender					
Male	192	76	116	Ref	
Female	196	78	118	0.859 (0.54–1.38)	0.528
Age groups, year					
Less than 34	101	40	61	Ref	
35 to 43	142	58	84	1.057 (0.48–2.32)	0.891
More than 44	145	56	89	1.017 (0.58–1.78)	0.954
Marital status					
Single	234	88	146	Ref	
Married	84	32	52	1.344 (0.74–2.44)	0.333
Divorce/widowed	70	34	36	1.409 (0.59–3.33)	0.433
Nationality					
Saudi	300	101	199	Ref	
Non-Saudi	88	53	35	2.434 (1.23–3.82)	0.010
Education					
Diploma	59	18	41	Ref	
Bachelor	187	75	112	2.104 (0.77–5.76)	0.148
Master	79	36	43	1.059 (0.45–2.49)	0.895
MD/PhD/Saudi Board	63	25	38	0.874 (0.37–2.07)	0.578
HCPs’ category					
Physicians and dentists	71	29	42	Ref	
Nurse	143	57	42	0.800 (0.31–2.07)	0.645
Pharmacists	82	33	86	0.842 (0.38–1.86)	0.672
Lab technicians	53	21	49	0.911 (0.40–2.08)	0.825
Other	39	14	32	0.798 (0.32–1.98)	0.626
Work experience					
Less than 5 years	93	34	59	Ref	
5 to 10 years	125	57	68	0.923 (0.38–2.24)	0.859
11 to 15	97	38	59	0.750 (0.38–2.24)	0.427
More than 15 years	73	25	48	0.893 (0.45–1.79)	0.750
Work Setting					
PHC	94	39	55	Ref	
General hospital	179	73	106	0.743 (0.39–1.43)	0.375
Specialty	115	42	73	0.918 (0.53–1.59)	0.761
Presence of chronic disease-DM					
No	276	102	174	Ref	
Yes	112	52	60	1.755 (1.15–2.63)	0.032

## Data Availability

The data presented in this study are available within the article and its Supplementary Materials. The raw data are not publicly available due to ethical restrictions involving human participants but can be made available from the corresponding author upon reasonable request.

## Supplementary Materials

The following supporting information can be downloaded at https://www.mdpi.com/article/10.3390/healthcare14020243/s1. File S1: WHO QoL Final.
